# Identification of enhancers responsible for the coordinated expression of myosin heavy chain isoforms in skeletal muscle

**DOI:** 10.1186/s12864-022-08737-9

**Published:** 2022-07-17

**Authors:** Keren Long, Duo Su, Xiaokai Li, Hengkuan Li, Sha Zeng, Yu Zhang, Zhining Zhong, Yu Lin, Xuemin Li, Lu Lu, Long Jin, Jideng Ma, Qianzi Tang, Mingzhou Li

**Affiliations:** Institute of Animal Genetics and Breeding, College of Animal Science and Technology, Sichuan Agricultural University, Chengdu, 611130 China

**Keywords:** *Myh* genes, Chromatin interaction, 4C-seq, Enhancer

## Abstract

**Background:**

Skeletal muscles consist of fibers of differing contractility and metabolic properties, which are primarily determined by the content of myosin heavy chain (MYH) isoforms (MYH7, MYH2, MYH1, and MYH4). The regulation of *Myh* genes transcription depends on three-dimensional chromatin conformation interaction, but the mechanistic details remain to be determined.

**Results:**

In this study, we characterized the interaction profiles of *Myh* genes using 4C-seq (circular chromosome conformation capture coupled to high-throughput sequencing). The interaction profile of *Myh* genes changed between fast quadriceps and slow soleus muscles. Combining chromatin immunoprecipitation-sequencing (ChIP-seq) and transposase accessible chromatin with high-throughput sequencing (ATAC-seq), we found that a 38 kb intergenic region interacting simultaneously with fast *Myh* genes promoters controlled the coordinated expression of fast *Myh* genes. We also identified four active enhancers of *Myh7*, and revealed that binding of MYOG and MYOD increased the activity of *Myh7* enhancers.

**Conclusions:**

This study provides new insight into the chromatin interactions that regulate *Myh* genes expression.

**Supplementary Information:**

The online version contains supplementary material available at 10.1186/s12864-022-08737-9.

## Background

Skeletal muscle is the most abundant tissue in mammalian bodies, accounting for about 40% of body weight, and comprises a diverse group of tissues with highly diverse origins, shapes, and differential susceptibility to injuries, drugs, and diseases [1]. In mammals, skeletal muscles are composed of heterogeneous myofibers that possess different functional and metabolic properties. Myofibers can be classified into type I, type IIa, type IId/x and type IIb according to the content of myosin heavy chain (MYH) isoforms, which include MYH7, MYH2, MYH1 and MYH4. For example, fast quadriceps muscle is composed of myofibers containing MYH4, while slow soleus muscle consists of myofibers containing MYH7 [2, 3]. Each myofiber type possesses a unique gene-expression profile; however, the mechanism of *Myh* genes transcriptional regulation has not been fully elucidated.

Enhancers are primary *cis*-regulatory elements (CREs) that are found in intronic, exonic, or intergenic regions [4, 5]. Spatial interactions between enhancers and promoters play critical roles in governing functions in cell type- and condition-specific manners [6]. For example, Hirsch et al*.* found that the enhancers of the *TWIST1* gene were critical for mesoderm development, and homozygous deletion of *eTw5-7* enhancers reduced *TWIST1* expression in the limb bud and caused pre-axial polydactyly [7]. Shyamsunder et al*.* showed that an enhancer of the *Cebpe* gene was necessary for granulocyte differentiation. Its deletion resulted in decreased *Cebpe* expression and severely blocked granulocyte differentiation [8]. Dos Santos et al. demonstrate that the fast *Myh* genes super enhancers (SE) is responsible for the non-stochastic robust coordinated fast *Myh* genes expression in the hundreds of body myonuclei present in adult myofibers [9]. Recent studies have revealed the enhancer repertoires controlling the identity of skeletal muscles in vivo [10] and genomic enhancer elements associated with skeletal muscle metabolism [11]. However, detailed information of how promoter-enhancer interactions can accurately regulate the expression of *Myh* genes is not known.

Circular chromosome conformation capture coupled to high-throughput sequencing (4C-seq) and related technologies (3C, 5C and Hi-C) are potent methods for studying three-dimensional nuclear organization. 4C-seq is an unbiased “one-versus-all” approach used to detect all genomic regions interacting with a specific fragment of choice [12]. It can generate high-resolution contact profiles for selected genomic sites based on limited amounts of sequencing reads [13]. Meanwhile, the histone 3 lysine 27 acetylation (H3K27ac) chromatin modification is a marker for active enhancers [14]. Additionally, assay for transposase-accessible chromatin combined with high-throughput sequencing (ATAC-seq) is an effective way to reveal chromatin accessibility at a genome-wide level [15]. The combination of epigenetic hallmarks (H3K27ac) and chromatin accessibility (ATAC-seq) can be used to screen for candidate active enhancers. In this study, we characterized the interaction profile of *Myh* genes between oxidative soleus and glycolytic quadriceps muscles. In addition, we identified active enhancers regulating *Myh* genes transcription. Our findings add to the knowledge of *Myh* genes regulation.

## Results

### Characterization of *Myh *genes interaction profiles in quadriceps and soleus muscles

**Fig. 1:**
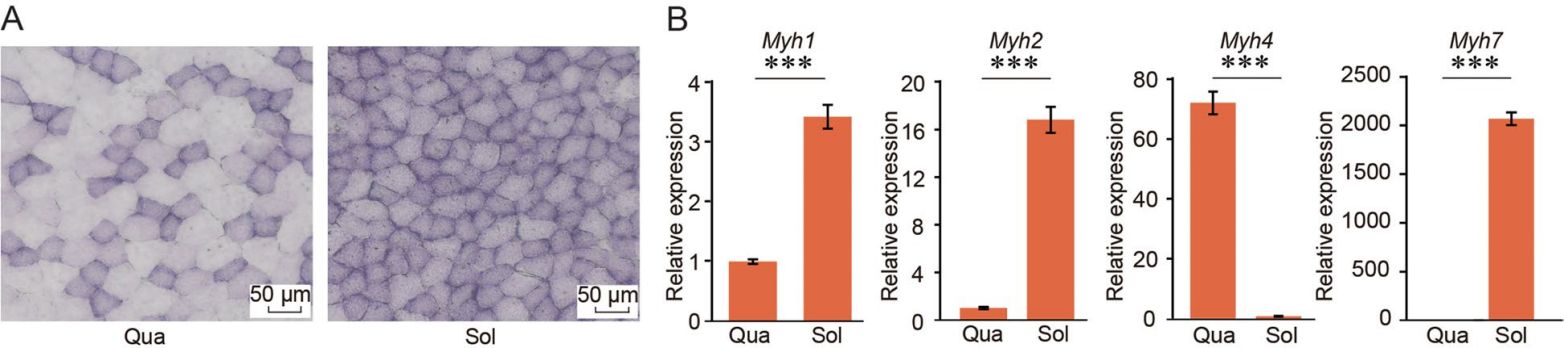
Interactomes identification of *Myh* genes in quadriceps and soleus. **A** Succinate dehydrogenase (SDH) staining of sections from quadriceps and soleus muscles (Scale bar represents 50 μm); **B** Relative expression levels of *Myh* isoforms measured in quadriceps and soleus (*n* = 3/group). Relative expression levels of genes were normalized to *Gapdh*. Data are represented as means ± SEM. ***p < 0.001

Oxidative soleus muscle had a high percentage of type I myofibers, and glycolytic quadriceps muscle tended to have a high percentage of type IIb myofibers (Fig. 1A). Consistent with previous studies, we detected significantly higher levels of *Myh7* and *Myh2*, and *Myh1* and lower levels of *Myh4* expression in soleus muscle compared with quadriceps muscle (Fig. 1B), indicating the varied expression of *Myh* genes between soleus and quadriceps muscles. To identify the genome-wide interacting pattern of *Myh* genes in fast quadriceps and slow soleus muscles, 4C-seq assays were performed using *Myh* genes promoters as the specific genomic region of interest, termed the 'viewpoint'. We constructed 16 4C-seq libraries for four different viewpoints (*Myh7*, *Myh2*, *Myh1* and *Myh4*) from soleus and quadriceps muscles in two biological replicates. We obtained approximately 42 million filtered reads, with an average of 2.6 million reads for each 4C-seq data, and approximately 18%–68% of the total reads were distributed on the viewpoint chromosome. Seventy-five percent (12/16) of the 4C datasets conformed to the “*cis*/overall ratio of > 40%” criteria (Additional file 1: Table S1) [16]. We then assessed Pearson’s correlation between biological replicates by counting the number of intrachromosomal interaction sites in every 1 Mb bin. As a result, all eight 4C experiment groups showed a Pearson’s correlation coefficient greater than 0.4 (Additional file 7: Fig. S1), indicating acceptable 4C-seq datasets.

We performed r3Cseq analysis [17] to identify genome-wide interaction sites. To minimize noise from random collisions within the nucleus and to identify reliable interaction sites, only overlapping regions shared between replicates were retained. As a result, only 3%–22% of interacting sites were reproducibly identified in the two biological replicates, reflecting the complexity of chromosome conformation interactions between nuclei (Additional file 2: Table S2). We thus focused on interaction sites that are reproducibly identified in both biological replicates, and these sites were considered to be high-fidelity interacting sites.

### Differences in *Myh* genes chromatin interactions between quadriceps and soleus muscles

**Fig. 2:**
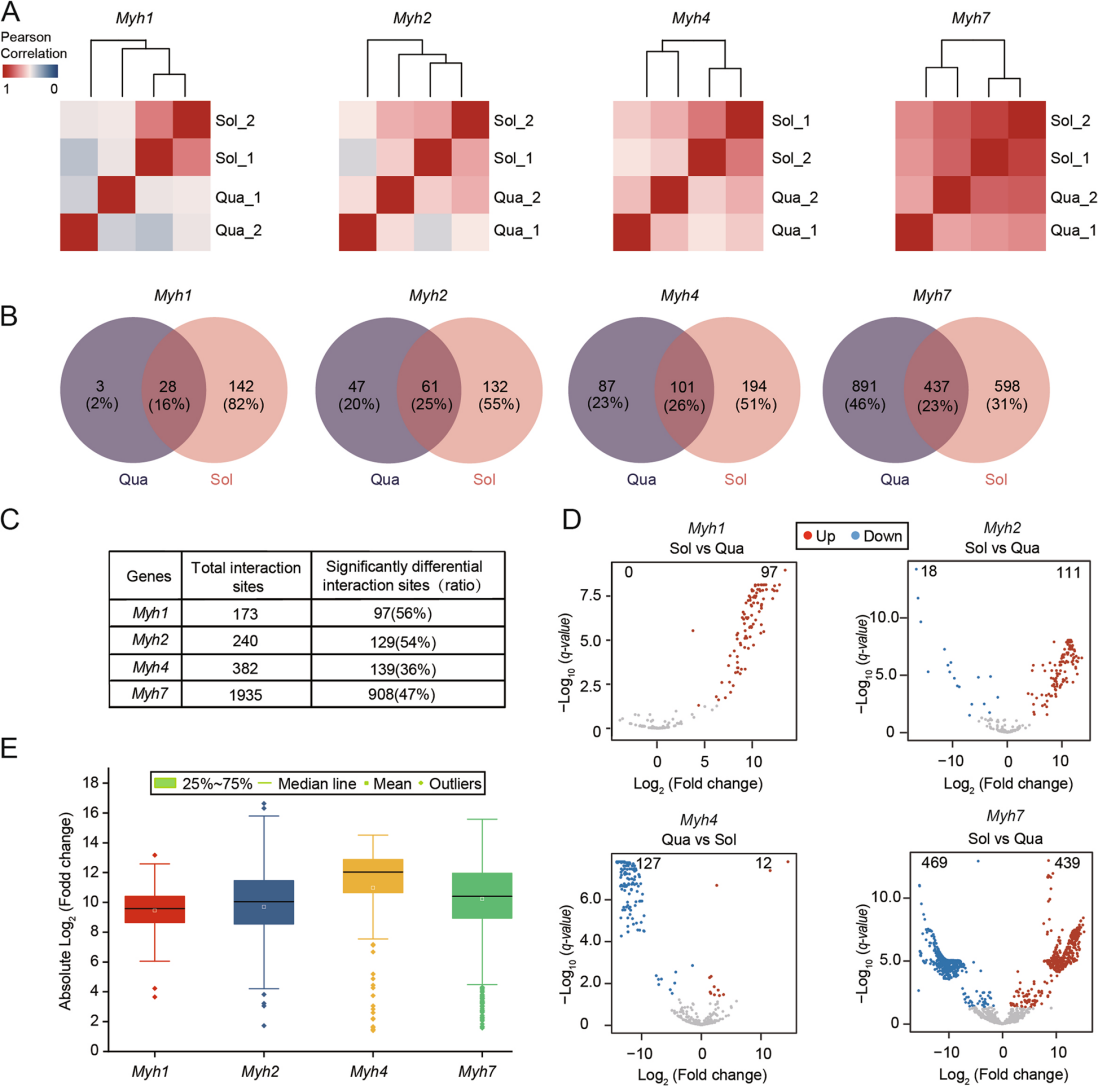
Chromatin interaction alterations of *Myh* genes between quadriceps and soleus. **A** Heatmap showing the clustering of *Myh* genes interactions in quadriceps and soleus. The color scale indicates the degree of correlation (blue, low correlation; red, high correlation). The clustering was generated using the Pearson correlation coefficient of interaction sites every 1 Mb bin; **B** A Venn diagram showing the number of common and unique genome-wide interactions site of the *Myh* genes between quadriceps and soleus; **C** Differential analysis of interaction sites identified reproducibly in two biological replicates; **D** Volcano plot of significantly differential interaction sites (SDISs) of *Myh* genes. The threshold of SDISs in the volcano plot was absolute log_2_Fold Change > 1 and padj < 0.05. Red and blue indicate significantly differential interaction sites; **E** The average absolute log_2_Fold Change of SDISs of the *Myh* genes

To investigate differences in chromatin conformation of *Myh* genes between quadriceps and soleus muscles, we performed hierarchical cluster analysis for each gene based on the number of intrachromosomal interaction sites in every 1 Mb bin. Cluster analysis separated the *Myh* genes into two muscles (Fig. 2A). In addition, only 16%–26% of the interaction sites were shared between the quadriceps and soleus muscles (Fig. 2B and Additional file 8: Fig. S2), indicating the highly divergent interaction profiles between the two muscles.

To further clarify details of the interaction differences between quadriceps and soleus muscles, DESeq2 [18] analysis was used to identify differential interaction sites with adjusted *p-*value < 0.05. As a result, 36%–56% of interaction sites were identified to be significant differential interaction sites (Fig. 2C and D). In addition, the average absolute log_2_ Fold Change of significant differential interaction sites for the *Myh* genes (*Myh7*, *Myh2*, *Myh1* and *Myh4*) were 10.2, 9.7, 9.5 and 11.0, respectively (Fig. 2E). The average absolute log_2_ Fold Change of all differential interaction sites for the *Myh* genes (*Myh7*, *Myh2*, *Myh1* and *Myh4*) were 5.2, 5.8, 5.4 and 4.2, respectively (Additional file 9: Fig. S3). These results indicate that interaction sites and interaction intensity of *Myh* genes changed between quadriceps and soleus muscles.

### A candidate enhancer region coordinated the expression of the fast *Myh* genes

**Fig. 3:**
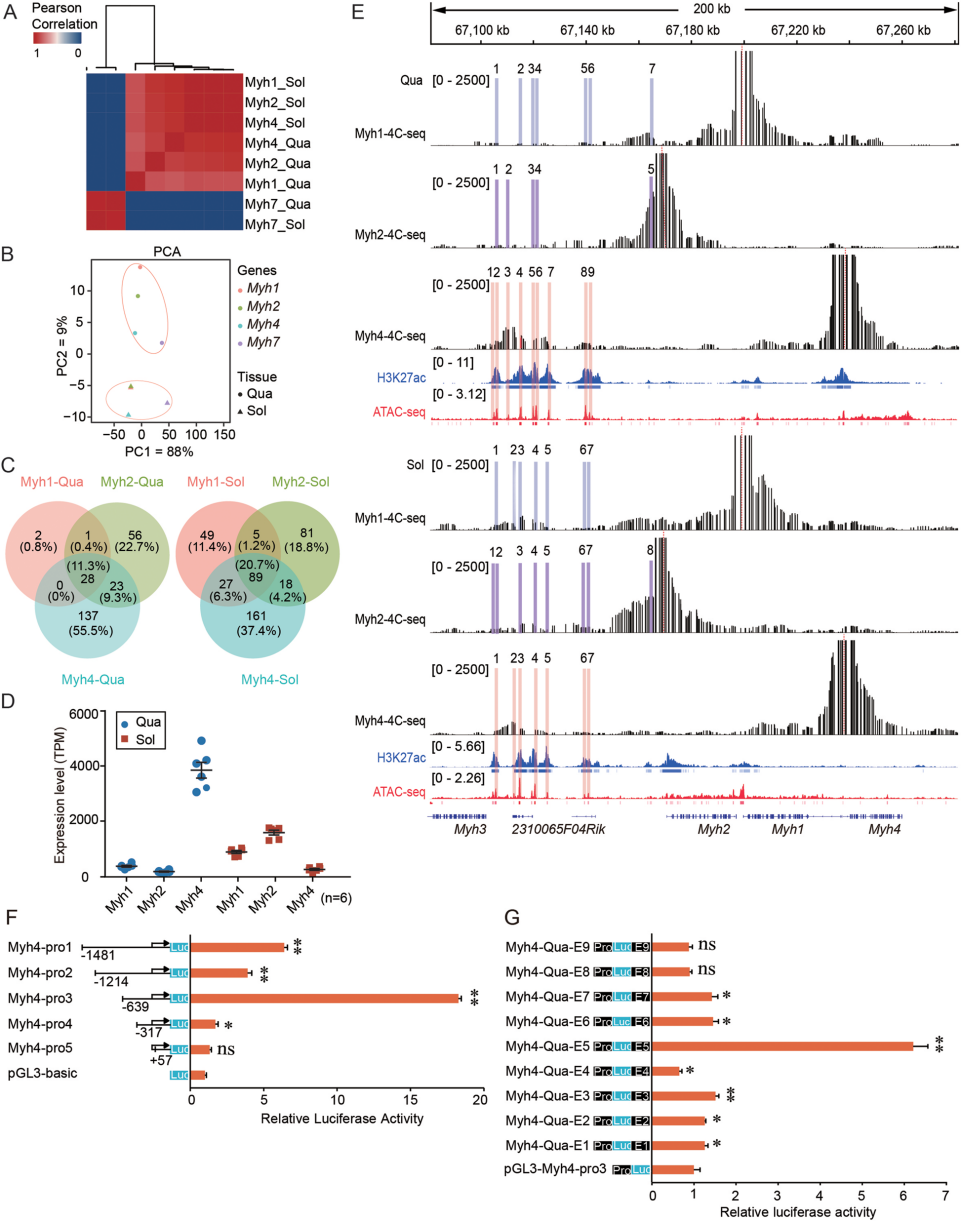
Identification and evaluation of candidate activity enhancers. **A** Heatmap showing the clustering of *Myh* genes interactions in quadriceps and soleus. The color scale indicates the degree of correlation (blue, low correlation; red, high correlation); **B** Principal component analysis (PCA) of *Myh* genes interactions in quadriceps and soleus. Each point represents a sample. The percentages on each axis represent the percentages of variation explained by the principal components. The clustering and PCA were generated using Pearson correlation coefficients for every 1 Mb bin cis-interaction site after merging the 4C sites of the respective biological replicates; **C** A Venn diagram showing the number of common and unique genome-wide interactions site of the *Myh* genes in the quadriceps and soleus; **D** RNA-seq gene expression for the fast *Myh* genes; **E** Candidate enhancers of fast *Myh* genes in quadriceps and soleus. 4C-seq interaction profile (black), ChIP-seq profiles for H3K27ac (blue), and ATAC-seq (red). The vertical dashed red line indicates the 4C viewpoint. Light bars indicate candidate enhancer regions. The blue and red vertical lines below the ChIP-seq and ATAC-seq profiles indicate the peak; **F** The relative promoter activity of different *Myh4* promoter regions was evaluated by luciferase reporter assay in H293 T cells. The pGL3-Basic was used as a control; **G** Dual-luciferase reporter assays to determine *Myh4* candidate enhancer activity in H293 T cells. The pGL3-Myh4-pro3 was used as a control. Data are represented as mean ± SD of three independent experiments, and *p-*values are calculated using Student’s t-test (*P < 0.05; **P < 0.01; ***P < 0.001)

The fast *Myh* genes, *Myh2*, *Myh4*, and *Myh1*, are located next to each other on chromosome 11 [19], and show robust coordinated expression in skeletal muscles [20]. To reveal the interactome profiles of these clustered fast *Myh* genes in different skeletal muscles, we performed hierarchical clustering analysis using a *cis*-interaction count in every 1 Mb bin. As a result, the slow gene, *Myh7*, showed a clear split from the three clustered fast genes (*Myh2, Myh4, Myh1*), consistent with the fact that *Myh7* is on another chromosome (Fig. 3A). The fast *Myh* genes always clustered together independent of tissue, indicating the critical roles of the local microenvironment of chromatin organization. PCA analysis produced similar results (Fig. 3B). Interestingly, the fast *Myh* genes showed a higher similarity in the slow soleus muscle (mainly expressing *Myh7*) than in the fast quadriceps muscle (mainly expressing the three clustered fast genes). Consistent with this, 20.7% of interaction sites were shared among three genes in the soleus muscle, but only 11.3% were shared in the quadriceps muscle (Fig. 3C). We downloaded published RNA-seq data [21] and observed greater differences in expression level across the fast *Myh* genes in quadriceps than in soleus (Fig. 3D), consistent with the chromatin interaction profiles of the fast *Myh* genes being more similar in soleus than in quadriceps muscle.

To further identify and compare the candidate enhancers regulating fast *Myh* genes, we manually annotated candidate active enhancers by combining 4C-seq data with chromatin immunoprecipitation-sequencing (ChIP-seq) and ATAC-seq peaks (see Materials and methods). We identified 21 and 22 candidate enhancers of fast *Myh* genes in quadriceps and soleus muscles, respectively. Overall, relatively more candidate active enhancers were observed in tissues with high levels of *Myh2* and *Myh4* expression, consistent with previous reports showing a positive correlation between gene expression level and the number of interacting enhancers [22].

Non-coding elements often display evolutionary conservation among organisms and regulate gene expression during ontogeny [23, 24]. Therefore, we analyzed the sequence conservation of the candidate active enhancer sequences across 60 vertebrates through the UCSC genome browser (http://genome-asia.ucsc.edu/). As a result, 93% (40/43) of the candidate active enhancers contained at least one conserved element (Additional file 3: Table S3), indicating high confidence of the candidate active enhancers.

Interestingly, 93% (40/43) of the potential enhancers identified were located in a 38 kb intergenic region (chr11: 67,104,519–67,142,456). This region shows strong enrichment of the active histone mark, H3K27ac, chromatin accessibility, and interacts simultaneously with the fast *Myh* genes promoters (Fig. 3E). We compared the interaction intensity of the fast *Myh* genes between the two muscles. We observed significantly more interactions between the *Myh4* promoter and the 38 kb intergenic region in quadriceps muscle, where *Myh4* is more transcribed than in the soleus. In contrast, we observed significantly more interactions between *Myh2* and *Myh1* promoters and the 38 kb intergenic region in soleus muscle, where *Myh2* and *Myh1* are more transcribed than in the quadriceps. In quadriceps muscle, which predominately expresses *Myh4*, strong and specific interactions between the 38 kb intergenic region and the *Myh4* promoter were observed. In soleus muscle, which mainly expresses *Myh7*, we observed no difference in the interaction intensity between the 38 kb intergenic region and the fast *Myh* genes promoters (Fig. 3E). These results showed that the 38 kb intergenic region forms chromatin loops with the promoters of the three clustered fast *Myh* genes, with three-dimensional spatial proximity directly coinciding with differential promoter activity in different fiber types.

Increasing gene transcription is the most significant feature of enhancers. The core gene promoter is the major determinant of promoter activity and gene expression [25, 26]. To further confirm candidate enhancer activity of the 38 kb intergenic region between *Myh3* and *Myh2*, we evaluated the activity of candidate enhancers with strong interactions with promoters in quadriceps muscle, which mainly expresses *Myh4*. We selected nine candidate active enhancers for luciferase reporter assays. We first identified the essential promoter region for *Myh4* transcription activation. Luciferase vectors driven by serial deletions of *Myh4* promoter were constructed and co-transfected with pRL-TK into H293T cells. Myh4-pro3 (-639 to + 385) showed the highest relative luciferase activity compared with the other fragments (*P* < 0.01) (Fig. 3F), indicating that it is essential to the promoter activity of *Myh4*. We then cloned each *Myh4* candidate active enhancer region into the pGL3-Myh4-pro3 reporter vector and determined luciferase activity. Compared with the control vectors, the Myh4-Qua-E3 and Myh4-Qua-E5 fragments showed significantly increased luciferase activity (*P* < 0.01) and Myh4-Qua-E5 had the most robust transcription activity (~ 6.2-fold higher compared with the empty vector) (Fig. 3G).

### Myogenic regulatory factors MYOG and MYOD increase enhancer activity on *Myh7*

**Fig. 4:**
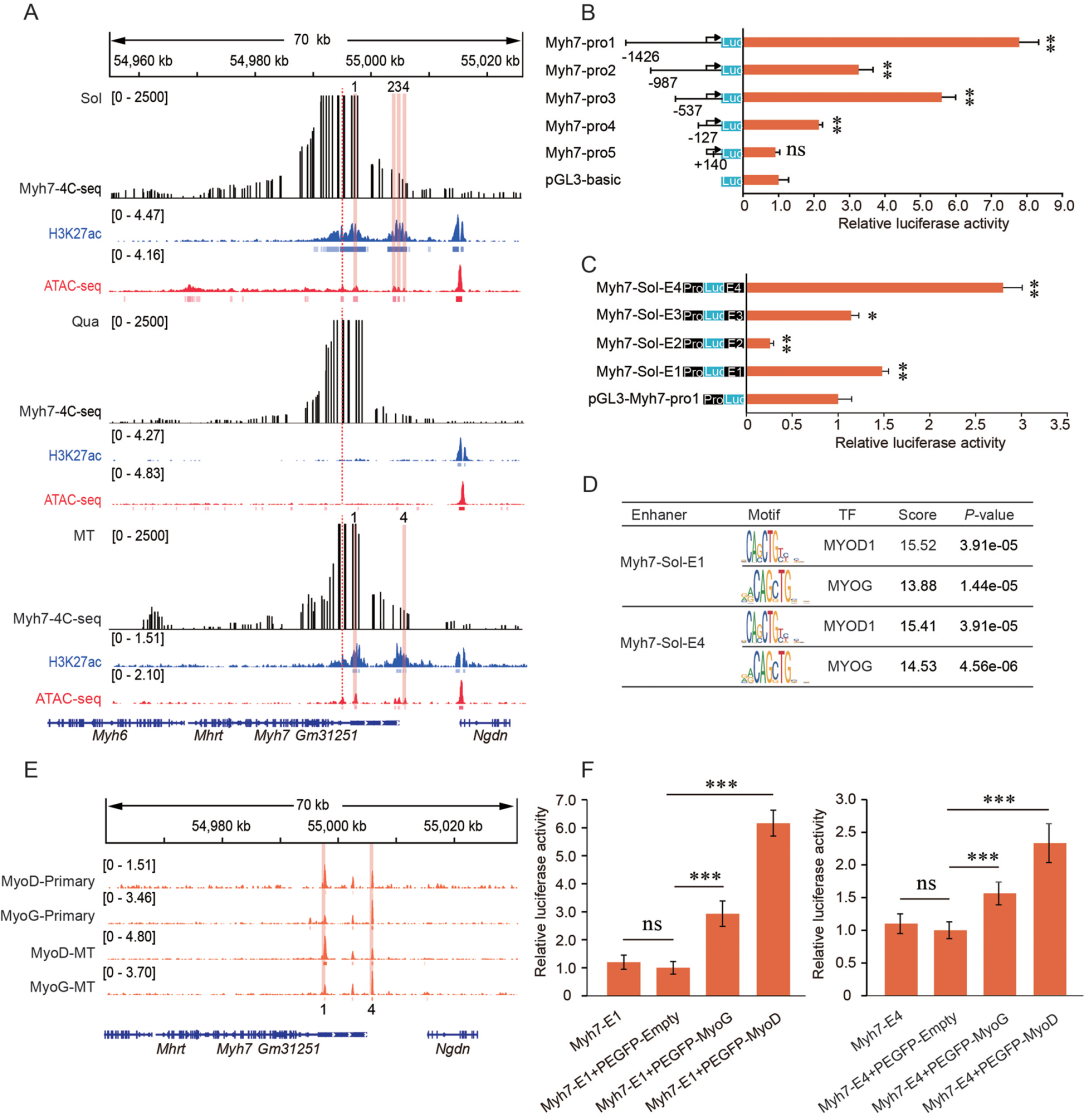
Binding of transcription factors at enhancers of *Myh7* in soleus. **A** Active enhancers *Myh7* in quadriceps, soleus and C2C12. 4C-seq interaction profile (black), ChIP-seq profiles for H3K27ac (blue), and ATAC-seq (red). The vertical dashed red line indicates the 4C viewpoint, and light red bars indicate candidate enhancer regions. The blue and red vertical lines below the ChIP-seq and ATAC-seq profiles indicate the peak; **B** The relative promoter activity of different *Myh7* promoter regions was evaluated by luciferase reporter assay in H293 T cells. The pGL3-Basic was used as a control; **C** Dual-luciferase reporter assays to determine *Myh7* candidate enhancer activity in H293 T cells. The pGL3-Myh7-pro1 was used as a control. **D** TF binding to the Myh7-Sol-E1, Myh7-Sol-E4 were predicted by JASPAR and AnimalTFDB3.0 website; **E** Public C2C12 myotubes and primary skeletal muscle cells ChIP-seq data were analyzed to show profiles of transcription factors in the Myh7-Sol-E1 and Myh7-Sol-E4 region. Light red bars indicate *Myh7* active enhancer regions; **F** pGL3-Myh7-promoter reporter constructs containing the Myh7-Sol-E1 or Myh7-Sol-E4 region were transfected into H293 T cells with control or TF overexpression, then luciferase activity was determined. Data are represented as mean ± SD of three independent experiments, and *p*-values are calculated using Student’s t-test (*P < 0.05; **P < 0.01; ***P < 0.001)

*Myh7* encodes the slow myosin heavy chain subtype (MyHCI) and is mainly expressed in the soleus muscle. However, few studies have investigated enhancers in *Myh7* gene transcription [27]. According to the enhancer screening strategy (see Materials and methods), we identified four candidate enhancers of 300–800 bp in the 10 kb upstream of the *Myh7* gene that were active in soleus, but not quadriceps, muscle. Visual inspection of the genomic profile showed that the active histone marker, H3K27ac, and chromatin accessibility, which are strongly associated with transcriptional activity, were strongly enriched in *Myh7*-expressing soleus muscle compared with *Myh7* non-expressing quadriceps muscle (Fig. 4A). Similar to the description above, we first identified the essential *Myh7* promoter region for transcription activation using luciferase reporter assays before evaluating the candidate *Myh7* enhancers that are active in soleus muscle. Myh7-pro1 (-1426 to + 283) showed the highest relative luciferase activity compared with other fragments (*P* < 0.01) (Fig. 4B). We then cloned each *Myh7* candidate active enhancer region into the luciferase reporter vector, pGL3-Myh7-pro1. Myh7-Sol-E1 and Myh7-Sol-E4 fragments showed significantly increased (*P* < 0.01) luciferase activity compared with the control vectors, and Myh7-Sol-E4 had the most robust transcription activity (~ 2.8-fold higher compared with the empty vector) (Fig. 4C).

Mouse C2C12 myoblasts can be induced to differentiate into C2C12 myotubes (C2C12-MTs) under in vitro culture conditions, and this cell line is widely used to investigate the molecular biology of muscle development. Therefore, we performed 4C-seq experiments with the *Myh7* promoter as viewpoints in C2C12-MTs. Consistently, Myh7-Sol 4C-seq interaction peaks and activity enhancers were also present in C2C12-MTs (Fig. 4A), indicating that active enhancers Myh7-Sol-E1 and Myh7-Sol-E4 have conserved biological functions in vivo and in vitro.

Myogenic regulatory factors (MRFs) are critical regulators of vertebrate skeletal muscle genes during early and adult myogenesis by binding to sequence-specific DNA elements (E-box, CANNTG) in the promoters of muscle genes. [28, 29]. Recent studies reported that transcription factors are recruited to specific enhancers to regulate gene expression [30]. The enhancers then act as a platform that supplies sufficient binding sites for various TFs in a stage‐specific manner [31]. Therefore, we used JASPAR [32] and AnimalTFDB3.0 [33] tools to identify MRFs that may bind to Myh7-Sol-E1 and Myh7-Sol-E4. We found significant enrichment of MYOG and MYOD motifs (Fig. 4D). The top 10 motifs enriched scores at Myh7-Sol-E1 and Myh7-Sol-E4 regions are shown in Additional file 10: Fig. S4.

To verify the binding of MYOG and MYOD to the above enhancer regions, we analyzed available mouse myoblast cell line and primary skeletal muscle cell ChIP-seq datasets for myogenesis-specific factors (MYOG and MYOD). We also observed that MYOG and MYOD bind strongly to Myh7-Sol-E1 and Myh7-Sol-E4 in C2C12-MTs and primary skeletal muscle cells (Fig. 4E). These findings indicate that MYOG and MYOD bind to Myh7-Sol-E1 and Myh7-Sol-E4, possibly to regulate *Myh7* expression.

To assess the effect of MYOG and MYOD on the activity of the above enhancers, we determined the luciferase activity of the Myh7-Sol-E1 and Myh7-Sol-E4 constructs after MYOG and MYOD overexpression in H293 T cells. Luciferase reporter assays showed a significant increase in luciferase activity of the Myh7-Sol-E1 and Myh7-Sol-E4 constructs after overexpression of MYOG and MYOD (Fig. 4F), indicating that MYOG and MYOD binding promoted the activity of Myh7-Sol-E1 and Myh7-Sol-E4. Together, our data indicate that binding of MYOG and MYOD to the Myh7-Sol-E1 and Myh7-Sol-E4 elements plays a critical role in regulating target gene expression.

## Discussion

Accumulating evidence demonstrates the importance of three-dimensional genome organization in the spatiotemporal regulation of gene expression [34, 35]. Using a high-resolution 4C-seq method with a relatively low amount of sequencing data [36], we mapped the interaction profile of four *Myh* genes in oxidative soleus and glycolytic quadriceps muscles. *Myh4* and *Myh7* showed greater average absolute log_2_ Fold Change of significant differential interaction sites between the two muscles. *Myh4* encodes the fastest muscle myosin heavy chain (MyHCIIb) and is mainly expressed in the quadriceps muscle, while *Myh7*, encoding the slow myosin heavy chain subtype (MyHCI), is mainly expressed in the soleus muscle. Consistently, differential analysis of publicly available RNA-seq data showed that the absolute log_2_ Fold Change of *Myh1* and *Myh2* was 0.6 and 1.2, respectively, indicating that the expression levels of *Myh1* and *Myh2* were not significantly different in quadriceps and soleus muscles. However, the absolute log_2_ Fold Change of *Myh4* and *Myh7* was 5.7 and 4.1, respectively, indicating that the expression levels of *Myh4* and *Myh7* were significantly different between the two muscles. Moreover, enrichment of H3K4me2 (highly correlated with chromatin accessibility [37]) and H3K27 (reflects regulatory region activity [14]) around *Myh4* and *Myh7* genes was significantly different between quadriceps and soleus muscles [10].

In mammals, some genes clustered at particular genomic loci show coordinated expression to perform similar functions. Transcriptional activation of clustered genes is associated with a dynamic three-dimensional chromatin architecture at these sites [38]. For example, at the *β-globin* locus, common regulatory sequences called locus control regions (LCRs) dynamically interact with different promoters within the locus to activate individual globin isoforms [39]. The interaction between the 38 kb intergenic region and the fast *Myh* genes promoters shows similarity to the human *β-globin* locus [40]. We found that the 38 kb intergenic region interacts simultaneously with fast *Myh* genes promoters to control the coordinated expression of fast *Myh* genes. Recent studies have also shown that two adjacent genes can be expressed at specific times through shared enhancers [41]. We observed that the promoter and the 38 kb intergenic region had stronger interactions in tissues with high expression levels of fast *Myh* genes, consistent with previous findings. In quadriceps muscle, which predominately expresses *Myh4*, strong and specific interactions between the 38 kb intergenic region and the *Myh4* promoter were observed. In soleus muscle, which mainly expresses the *Myh7* gene, we observed approximately no difference in interaction intensity between the 38 kb intergenic region and the fast *Myh* genes promoters. These results indicate that the interaction between the *Myh4* promoter and the 38 kb intergenic region plays an important regulatory role in quadriceps muscle, which may affect fast myofiber formation. However, we have not yet elucidated which active enhancer or combination of active enhancers could be responsible for sequential and specific *Myh* genes expression in fast quadriceps and slow soleus muscles. In the mouse genome, an intergenic region 50 kb upstream of the *Myh2* gene and 4 kb upstream of a long intergenic noncoding RNA (lincRNA) (2310065F04Rik) was identified as *linc-MYH*, which coordinates fiber-type gene expression [42]. Recently, Dos Santos et al. found that the 42 kb intergenic region between *Myh3* and *Myh2* is a super-enhancer composed of multiple enhancer elements, and that the fast *Myh* genes promoters compete for the super-enhancer [9]. The 38 kb intergenic region we identified is contained within the 42 kb super-enhancer.

*Myh7* encodes the slow myosin heavy chain subtype (MyHCI) and is highly expressed in oxidative skeletal muscles. We identified four candidate active enhancers by combining 4C-seq with ChIP-seq and ATAC-seq peaks in soleus muscle. The function of muscle regulatory regions can be highly divergent in vitro and *in vi*vo [10]; however, here we revealed relatively similar interaction profiles for *Myh7* between soleus and C2C12-MT cells, indicating that these enhancers may be functionally conserved and play a crucial role in regulating the *Myh7* gene. Enhancers can act as integrated platforms for TF binding, leading to controlled cell/tissue-specific gene expression [43]. Enhancers contain a high-density DNA motifs recognized by specific TFs [31]. The MRFs are four muscle-specific proteins, MYOD, MYF5, MYOG, and MRF4, that can cooperate with MEF2 and bind to the E-box to induce muscle-specific gene expression [44, 45]. As the primary regulator of myogenesis, MYOD converts fibroblasts into myoblasts and promotes the formation of multinucleated myotubes [46]. MYOG plays a significant role at the later stage of myogenesis [47–49]. Knockdown of MYOG can reverse terminal muscle cell differentiation [50]. In vivo MYOG-null mutation results in the near-complete absence of myofibers [51]. TFs bind to promoters and enhancer regions, and recruit chromatin modifiers for activation or repression of cell-specific gene expression [52]. Our results show that MYOG and MYOD bind to Myh7-Sol-E1 and Myh7-Sol-E4. MYOG and MYOD are critical transcription factors in skeletal muscle differentiation [53–56]. Myogenesis is orchestrated through a series of transcriptional controls governed by MRFs. MYOD, a master gene for myogenesis, is expressed at an early stage of myogenic differentiation and induces the expression of MYOG and MRF4 [57]. They cooperate with the myocyte enhancer factor-2 family of TFs to activate the expression of most myogenesis-related genes and promote the differentiation of myoblasts [58–60]. MRFs not only act as activators during myogenesis, but also regulate the enhancer activity of muscle-specific genes. Blum et al. showed that approximately 80% of myotube-specific enhancers exhibit predicted MYOD binding sites [61].

Our results show that the luciferase activity of Myh7-Sol-E1 and Myh7-Sol-E4 constructs increased significantly after MYOG and MYOD overexpression, which indicates that the enhancer activity of Myh7-Sol-E1 and Myh7-Sol-E4 regions requires the binding of MYOG and MYOD. Whether these transcription factors are essential for the transcriptional activation of *Myh7* gene remains to be studied.

## Conclusions

In this study, we constructed high-resolution genome-wide interaction maps of *Myh* genes in mice oxidative soleus and glycolytic quadriceps muscles. We identified active enhancers of *Myh* genes and revealed the important roles of MYOG and MYOD in regulating *Myh7* gene expression.

## Materials and methods

### Ethics statement

All research involving animals was conducted according to Regulations for the Administration of Affairs Concerning Experimental Animals (Ministry of Science and Technology, China, revised in March 2017), and approved by the animal ethical and welfare committee (AEWC) of Sichuan Agricultural University under permit No. DKY-B2019202011. This study was carried out in compliance with the ARRIVE guidelines.

### Animals materials

Thirty male C57BL/6 J mice (7–8 weeks old) were purchased from Chengdu Dossy Experimental Animals Co., Ltd (Chengdu, China). All mice were euthanized, and their quadriceps and soleus muscles were rapidly isolated and stored in liquid nitrogen for subsequent experiments.

### Cell culture and C2C12 myoblast differentiation

C2C12 and H293T cells were kindly provided by the Cell Bank of the Chinese Academy of Sciences (Shanghai, China). 293FT cells were purchased from Thermo Fisher Scientific. Cells were grown in DMEM (Gibco, USA) supplemented with 10% FBS (Gibco, USA) and 1% penicillin–streptomycin (Gibco, USA) at 37℃ under 5% CO_2_. For the C2C12 induction of differentiation, 60–70% confluent cells were cultured in DMEM supplemented with 2% horse serum (HyClone, USA). The differentiation media were changed every two days.

### Histochemical SDH staining

Quadriceps (Quad) and soleus (Sol) of the hind limb were dissected and were coated in optimal cutting temperature (OCT) compound, then snap-frozen in liquid nitrogen-chilled isopentane. Muscles samples were sliced into 12-μm-thick cross-sections using a cryostat at -20 °C. The tissue sections were dried at room temperature for 15 min. SDH staining was performed manually in a solution containing nitroblue tetrazolium chloride (Solarbio, China), following the manufacturer’s instructions.

### RNA isolation and qRT-PCR analysis

Tissue total RNA was extracted using TRIzol reagent (Invitrogen, USA). For qRT-PCR, RNA was reverse-transcribed using HiScript III 1st Strand cDNA Synthesis Kit with gDNA wiper (Vazyme, China) following the manufacturer’s instructions. Quantitative RT-PCR (qRT-PCR) amplification was performed on a CFX96 Touch (Bio-Rad, USA) using the ChamQ Universal SYBR qPCR Master Mix (Vazyme, China). PCR amplification parameters were 95 °C (30 s) and 40 cycles of 95 °C (10 s), 60 °C (30 s). All samples were repeated in triplicate. Relative expression levels of mRNAs were calculated using the 2^−ΔΔCt^ method after normalization to mRNA expression of the housekeeping gene *Gapdh*. The student’s t-test was used for assessing significance (*p*-values). The primer sequences are listed in Additional file 4: Table S4.

### Circularized chromosome conformation capture (4C) assay and sequencing

4C libraries preparation was performed following the previously described protocol [13, 16] with some changes for primary tissue. Experiments for each gene were performed on two biological replicates of muscles samples from C57BL/6 mice, respectively. In brief, skeletal muscle was dissected and snap-frozen in liquid nitrogen and dissociated into single cells that were fixed with freshly prepared 2% formaldehyde for 10 min at RT. The fixation was quenched with cold glycine at a final concentration of 125 mM, and cells were lysed in 1 ml cold lysis buffer (50 mM Tris, pH7.5, 150 mM NaCl, 5 mM EDTA, 0.5% NP-40, 1% Triton X-100, protease inhibitors) incubate for 10 min on ice. Nuclei were pelleted by centrifugation and washed twice with PBS. Primary digestion of Nuclear DNA with DpnII (New England Biolabs) was performed overnight at 37˚C. Fragments were ligated with T4 DNA ligase overnight at 16˚C. Reverse crosslinking was carried out at 65˚C for 12 h with proteinase K, followed by RNase A digestion, phenol/chloroform extraction precipitation. DNA was further digested by Csp6 I (New England Biolabs) overnight, followed by proximity ligation and purification to obtain the 4C libraries. The 4C libraries were generated by performing a two-step PCR strategy. DNA was amplified using outer primers in the first PCR, and one-tenth of the first PCR product was used as a template in the second PCR using nested primers. PCR was performed using Phusion DNA polymerase (Thermo Scientific), with 3.5 μg of the template, and 14 individual PCR reactions were performed on 250 ng of 4C template each. After pooling, 4C samples were purified using VAHTS DNA Clean Beads (Vazyme, China) at a 1.5:1 ratio of beads to sample. Ten micrograms of the PCR products were size-selected on a 2% agarose gel (200–800 bp), and unwanted PCR product bands were removed. 4C libraries were sequenced using single-end 150 bp reads on an Illumina NovaSeq 6000 system. The outline of the 4C-seq procedure, viewpoint selection are shown in Additional file 11–12: Fig. S5-6. The primer sequences are listed in Additional file 5: Table S5.

### 4C-seq data analysis

4C-seq analysis was performed using the pipe4C pipeline [13] and R-package r3Cseq [17]. Briefly, trimmed reads from each replicate were mapped to the masked version of the reference mouse genome (masked for the gap, repetitive, an ambiguous sequences) downloaded from the R Bioconductor repository (BSgenome.Mmusculus.UCSC.mm10.masked) using bowtie2 (v2.4.2). Both for pipe4C pipeline and r3Cseq, reads corresponding to self-ligated or non-digested fragments were removed. The viewpoint chromosome and DpnII was used as the restriction enzyme to digest the genome. A non-overlapping window size of 2 kb was selected to identify interacted regions. The number of mapped reads for each window was counted and then normalized to obtain RPM (reads per million per window) values to perform statistical analysis. The significantly interacting regions (adjusted *p-*value < 0.05) were identified using r3Cseq. The raw read counts at each interaction site were used for differential analysis using the DESeq2 R package, where each interaction site is considered as a feature (or gene). The DESeq2 was used to find differentially interacting sites, defined as sites with an absolute log_2_ Fold Change greater than 1 and an adjusted *p*-value < 0.05.

### Public ChIP-seq and ATAC-seq data

Publically available ChIP-seq sequencing data of H3K27ac and ATAC-seq datasets for Quad and Sol were downloaded from the Gene Expression Omnibus (GEO) database under accession ID GSE123879. MYOG and MYOD ChIP-seq sequencing datasets for mouse myoblast cell line and primary skeletal muscle cell were downloaded from the GEO database under accession ID GSE49313 and GSE56077.

### Analysis of ChIP-seq and ATAC-seq data

ChIP-seq and ATAC-seq raw reads were aligned to the mouse mm10 genome using BWA (version 0.7.17). PCR duplicates of ChIP-seq and ATAC-seq data were removed with samtools and Picard (version 1.124), respectively. ChIP-seq peaks were called by macs2 (version 2.2.7.1) using the “–nomodel” parameter. Overlapping peaks were merged for replicate experiments before further analysis. The bigwig files were generated by bedGraphToBigWig (version 2.8), and were visualized in IGV.

### Screening of candidate activity enhancers

4C-seq interaction peak generated by pipe4C simultaneous enrichment with H3K27ac and ATAC-seq peaks eliminate annotated promoter and interchromosomal regions. We manually selected the position of the maximum signal (≤ 1000 bp) of ATAC-seq was defined as candidate active enhancers. The genomic coordinates of candidate active enhancers are shown in Additional file 3: Table S3.

### Plasmid constructs

The *Myh4* and *Myh7* promoter of a series of 5'—deletion and 3' fragments -1481/ + 385 (Myh4-pro1), -1214/ + 385 (Myh4-pro2), -639/ + 385 (Myh4-pro3), -317/ + 385 (Myh4-pro4), + 57/ + 385 (Myh4-pro5) and -1426/ + 283 (Myh7-pro1), -987/ + 283 (Myh7-pro2), -537/ + 283 (Myh7-pro3), -127/ + 283 (Myh7-pro4), + 140/ + 283 (Myh7-pro5), and candidates active enhancer sequence were amplified from mouse genomic DNA using 2 × Phanta Flash Master Mix (Vazyme, China). Each promoter fragment was ligated into a pGL3-Basic reporter vector (Promega) upstream of the luciferase gene, using KpnI and HindIII (NEB, USA) restriction sites. Subsequently, the candidate enhancer region was cloned into a pGL3-promoter reporter vector that contains a Firefly luciferase gene driven by the promoter with the highest relative luciferase activity.

The coding sequence (CDS) of MYOD and MYOG were amplified from cDNA using 2 × Phanta Flash Master Mix (Vazyme, China). Each CDS fragment was ligated into a pEGFP-N1 vector using HindIII (NEB, USA) restriction sites. The primer combinations used to construct each vector are shown in Additional file 6: Table S6.

### Luciferase reporter assays

All constructs were verified using Sanger sequencing and cotransfected with a Renilla luciferase control plasmid into H293T cells in quadruplicate. Transfection was performed using Lipofectamine™ 3000 (Thermo Fisher Scientific) following the manufacturer’s protocol. 36 h post-transfection Firefly and Renilla luciferase activity were determined using the dual-luciferase reporter assay kit (Promega) according to manufacturer’s instructions, on a Promega GloMax® 96 Microplate Luminometer. All of the relative luciferase activities were normalized to the same protein concentration.

### Statistical analysis

The Student t-test (two-tailed) was performed to determine the statistical significance of the experimental results. All data were expressed as mean ± SEM. *P* < 0.05 is considered statistically significant.

## Supplementary Information

**Additional file 1: Table S1.** Detailed quality metrics of each 4C data.

**Additional file 2: Table S2.** Summary of metrics in 4C-seq analysis in quadriceps and soleus.

**Additional file 3: Table S3.** The genomic coordinates and conserved element analysis.

**Additional file 4: Table S4.** The primer for RT-PCR.

**Additional file 5: Table S5.** The primer for 4C-seq library construction.

**Additional file 6: Table S6.** The PCR primers of constructed pGL3-basic and pEGFP-N1 vectors.

**Additional file 7: Figure S1.** Scatter plot showing interactions of *Myh* genes in quadriceps and soleus.

**Additional file 8: Figure S2.** Circos plots of genome-wide interaction sites of *Myh* genes.

**Additional file 9: Figure S3.** The average absolute log2Fold Change of all differential interaction sites of the *Myh* genes.

**Additional file 10: Figure S4.** Myh7-Sol-E1 and Myh7-Sol-E4 transcription factor enrichment analysis.

**Additional file 11: Figure S5.** Schematic workflow of the 4C-seq procedure.

**Additional file 12: Figure S6.** Schematic viewpoint selection of *Myh* genes.

## Data Availability

The sequencing results of 4C-seq data in our experiment are available in the NCBI Sequence Read Archive (SRA; https://www.ncbi.nlm.nih.gov/sra/) under accession numbers PRJNA795582.

## Abbreviations

*Myh*: Myosin heavy chain
4C-seq: Circular chromosome conformation capture coupled to high-throughput sequencing
ChIP-seq: Combining chromatin immunoprecipitation-sequencing
ATAC-seq: Transposase accessible chromatin with high-throughput sequencing
CREs: Cis-regulatory elements
H3K27ac: Histone 3 lysine 27 acetylation
SDH: Succinate dehydrogenase
MRFs: Myogenic regulatory factors
C2C12-MTs: C2C12 myotubes
