# Supplementary figures and images for: Identification of enhancers responsible for the coordinated expression of myosin heavy chain isoforms in skeletal muscle
Source: BMC Genomics. 2022 Jul 17;23:519. doi: 10.1186/s12864-022-08737-9 (PMC9288694; doi:10.1186/s12864-022-08737-9)

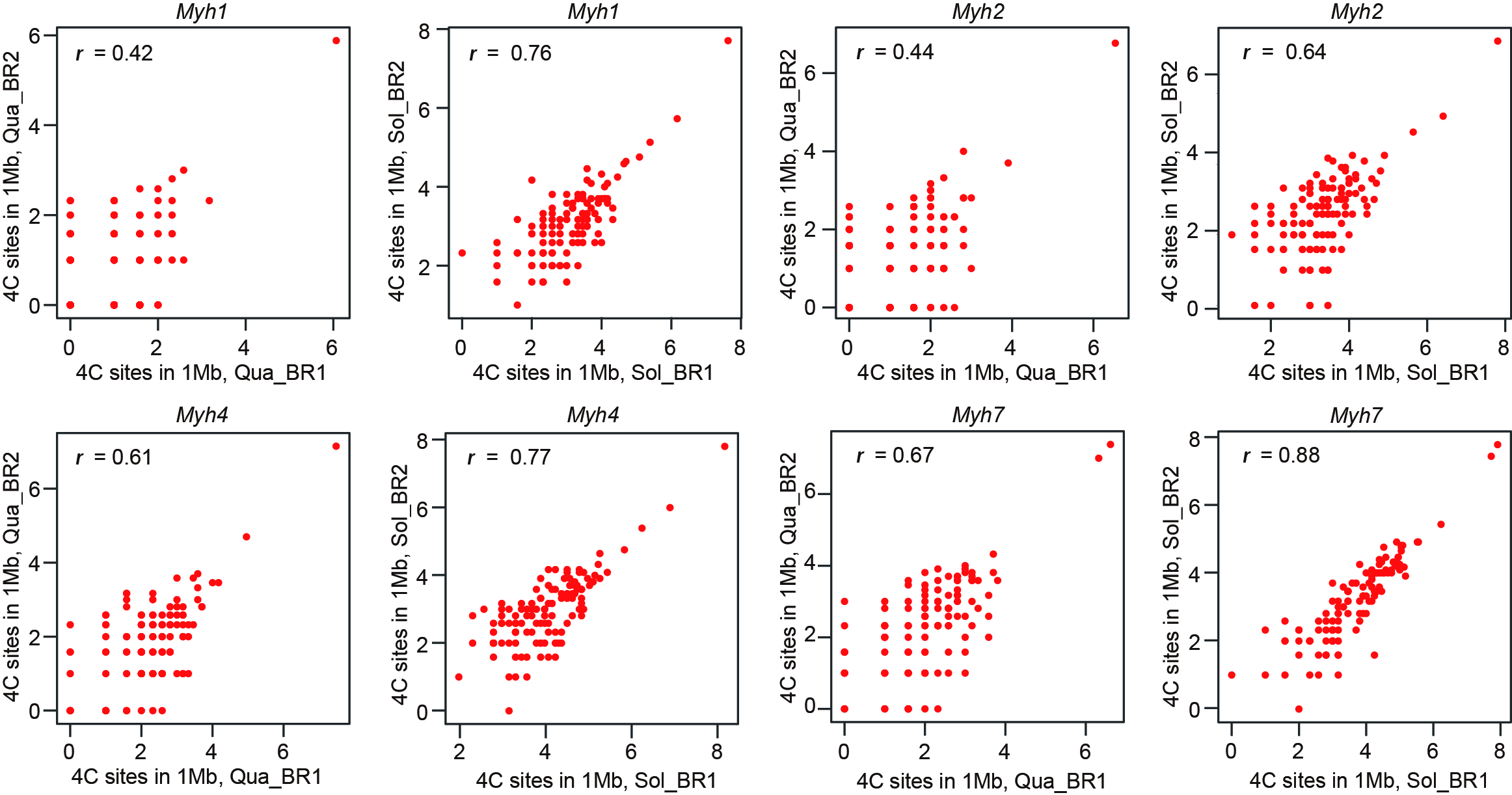

Supplement: Supplementary file 7 — Additional file 7: Figure S1. Scatter plot showing interactions of Myh genes in quadriceps and soleus. [file 12864_2022_8737_MOESM7_ESM.jpg]

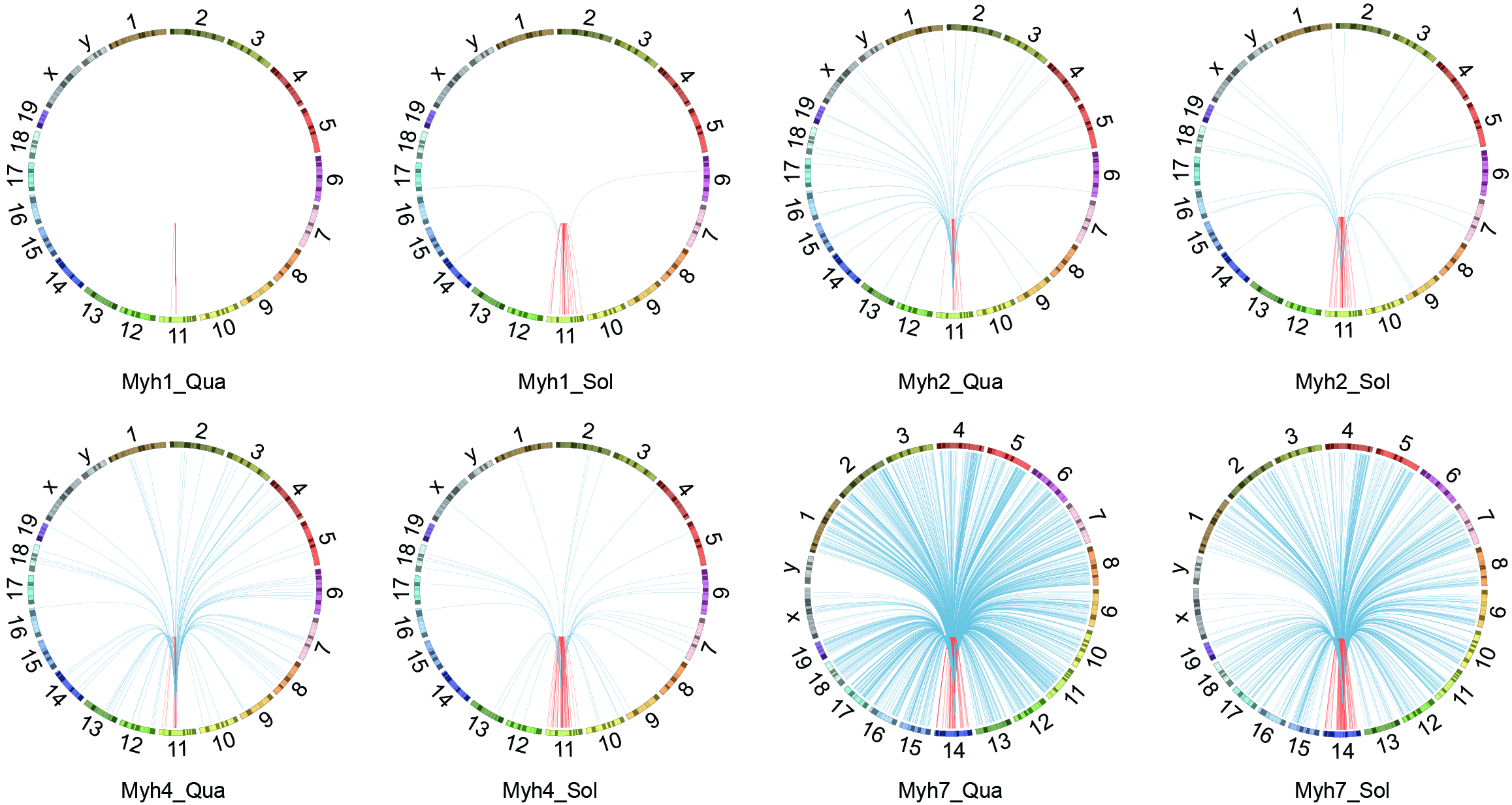

Supplement: Supplementary file 8 — Additional file 8: Figure S2. Circos plots of genome-wide interaction sites of Myh genes. [file 12864_2022_8737_MOESM8_ESM.jpg]

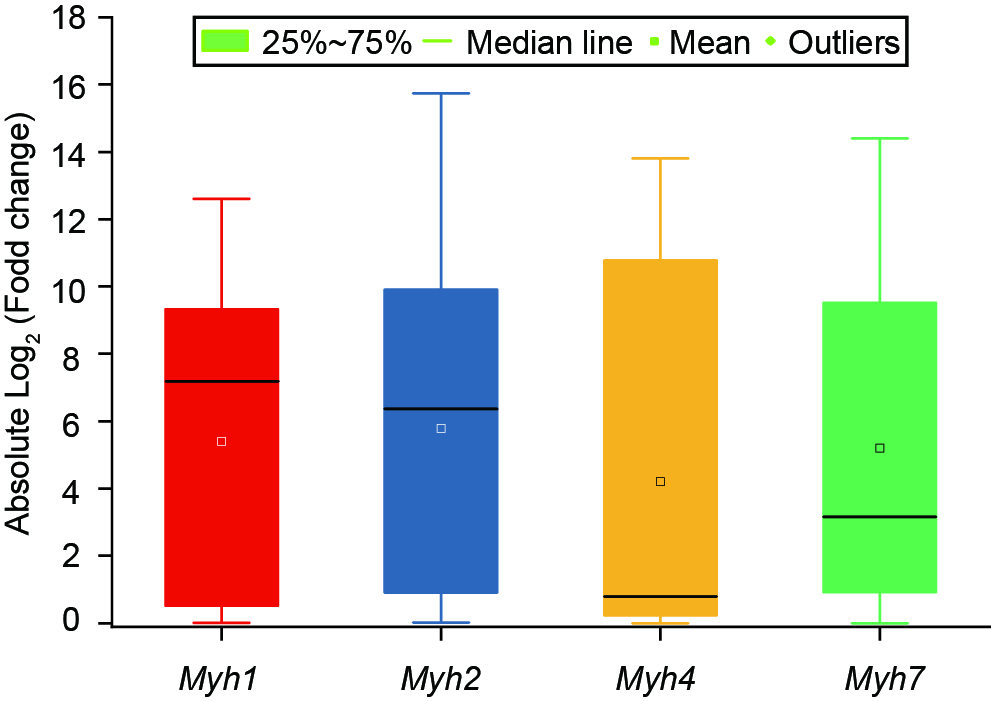

Supplement: Supplementary file 9 — Additional file 9: Figure S3. The average absolute log2Fold Change of all differential interaction sites of the Myh genes. [file 12864_2022_8737_MOESM9_ESM.jpg]

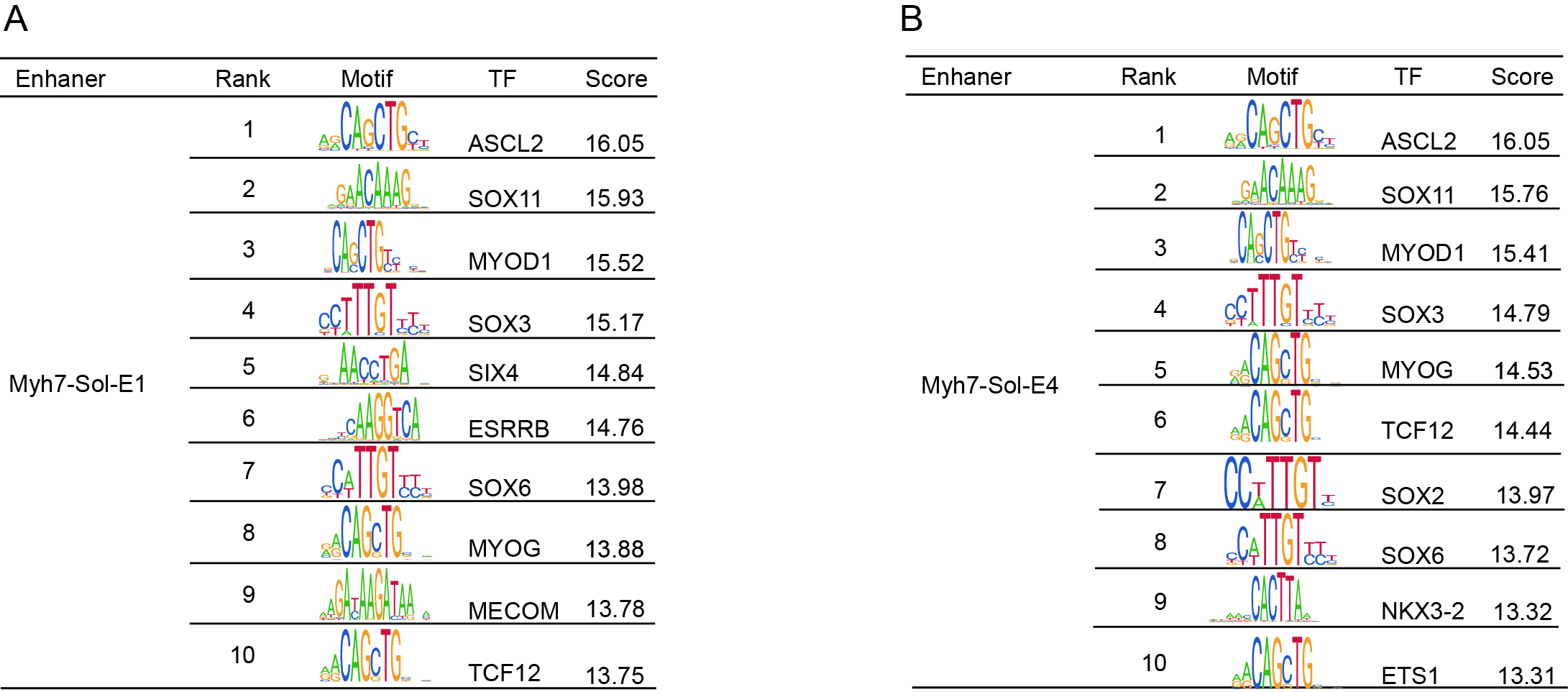

Supplement: Supplementary file 10 — Additional file 10: Figure S4. Myh7-Sol-E1 and Myh7-Sol-E4 transcription factor enrichment analysis. [file 12864_2022_8737_MOESM10_ESM.jpg]

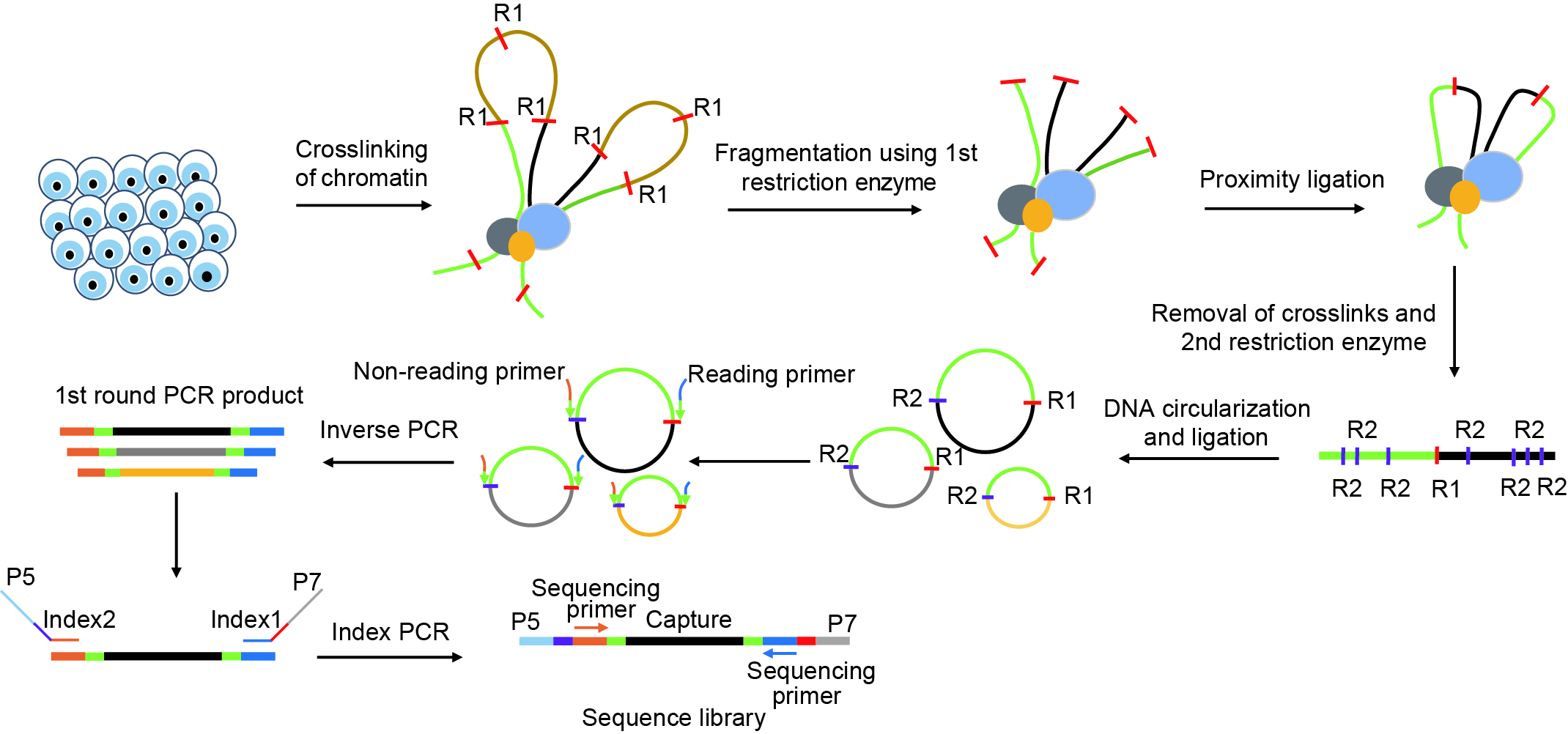

Supplement: Supplementary file 11 — Additional file 11: Figure S5. Schematic workflow of the 4C-seq procedure. [file 12864_2022_8737_MOESM11_ESM.jpg]

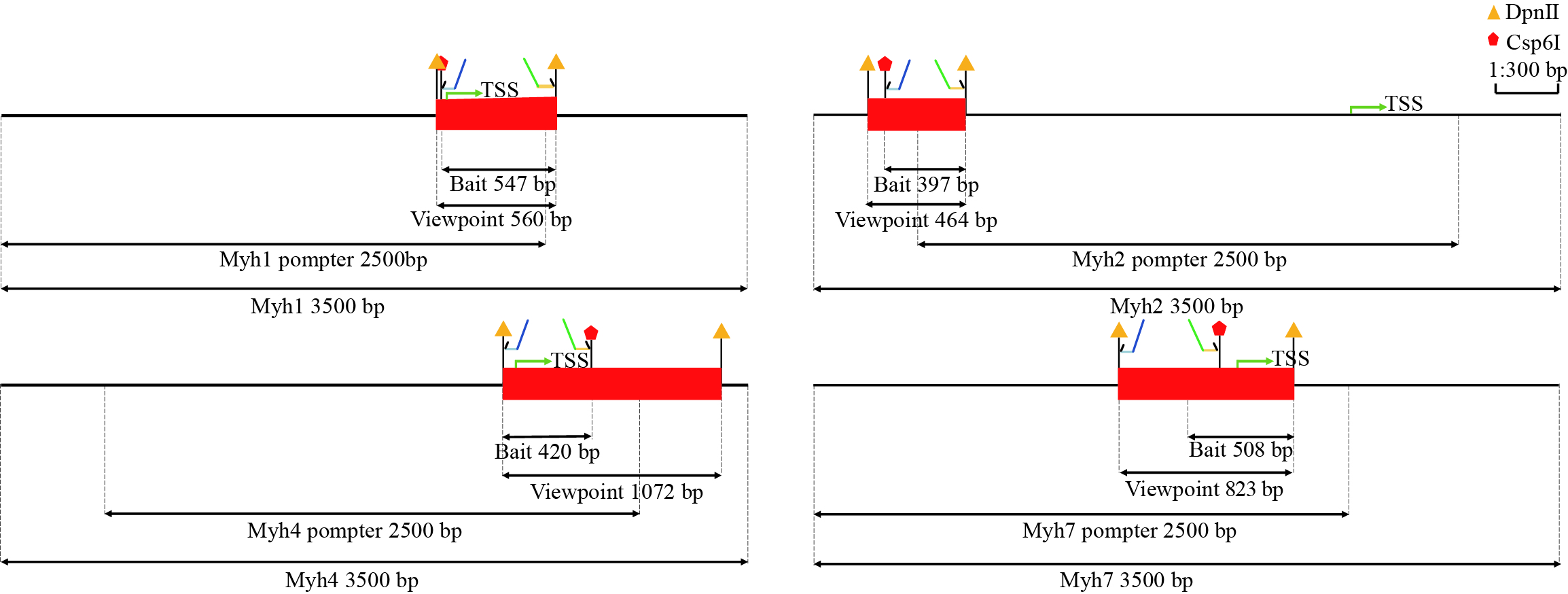

Supplement: Supplementary file 12 — Additional file 12: Figure S6. Schematic viewpoint selection of Myh genes. [file 12864_2022_8737_MOESM12_ESM.jpg]
